# Supplementary material for: Bond engineering of molecular ferroelectrics renders soft and high-performance piezoelectric energy harvesting materials
Source: Nat Commun. 2022 Sep 24;13:5607. doi: 10.1038/s41467-022-33325-6 (PMC9509372; doi:10.1038/s41467-022-33325-6)
Supplement: Supplementary file 1 — Supplementary Information [file 41467_2022_33325_MOESM1_ESM.pdf]

Supplementary Information for

# Bond engineering of molecular ferroelectrics renders soft and high-performance piezoelectric energy harvesting materials

Yuzhong Hu[1,2,*,┼], Kaushik Parida[3,4,┼], Hao Zhang[3], Xin Wang[5], Yongxin Li[6], Xinran Zhou[3], Samuel Alexander Morris[7], Weng Heng Liew[8], Haomin Wang[3], Tao Li[3], Feng Jiang[3], Mingmin Yang[2], Marin Alexe[2], Zehui Du[9], Chee Lip Gan[3], Kui Yao[8], Bin Xu[5], Pooi See Lee[3,*] and Hong Jin Fan[1,*]

[1]School of Physical and Mathematical Sciences, Nanyang Technological University, Singapore 637371

[2]Department of Physics, The University of Warwick, Coventry CV4 7AL, UK

[3]School of Materials Science and Engineering, Nanyang Technological University, Singapore 639798

[4]Department of Polymer and Process Engineering, Indian Institute of Technology, Roorkee, Uttarakhand 247667, India

[5]Jiangsu Key Laboratory of Thin Films, School of Physical Science and Technology, Soochow University, 1 Shizi Street, Suzhou 215006, China

[6]Division of Chemistry and Biological Chemistry, School of Physical and Mathematical Sciences, Nanyang Technological University, Singapore 637371

[7]Facility for Analysis, Characterisation, Testing and Simulation (FACTS), Nanyang Technological University, Singapore, 639798

[8]Institute of Materials Research and Engineering, A*STAR (Agency for Science, Technology and Research), Singapore.

[9]Temasek Laboratories, Nanyang Technological University, Singapore, 637553

┼These authors contributed equally to this work.

*Corresponding author. Email: huyu0012@e.ntu.edu.sg (Y.H.); pslee@ntu.edu.sg (P.S.L.); fanhj@ntu.edu.sg (H.J.F.)

## This PDF file includes:

Supplementary Text 1 – 3

Supplementary Figs. 1 –13

Supplementary Tables 1 –4

Supplementary References

## Table of Content

Supplementary Text 1 | Direct piezoelectricity measurements…………….……….4

Supplementary Text 2 | Converse piezoelectricity measurements…………………….5

Supplementary Text 3 | Energy harvesting measurements……………..……….….8

Supplementary Figures……….…………………………………………….……10

Supplementary Tables…………………….………………………………..…….24

Supplementary References……………………………………………………………29

# Supplementary Text

## Supplementary Text 1 | Direct piezoelectricity measurements

In 1950s, to investigate the piezoelectricity of perovskite oxide with obvious domain reorientation feature (PZT and BTO), Berlincourt established a mechanical system with tuneable stress amplitude and frequency to provide different outer stimuli[1]. This quasi-static piezoelectric measurement was then called Berlincourt method and the established facility is regarded as Berlincourt piezometer[2]. In this system, piezoelectrics is clamped with static force and excited by AC stress with frequency far below the resonant point. The produced charge from direct piezoelectric effect was recorded to obtain the $d_{33}$ value[1,2]. Following this method, we built a well calibrated piezometer during our previous work[3]. Under a constant electric field condition, the $d_{33}$ of the tested sample was obtained by:

$$d_{33} = \frac{\partial D_3}{\partial T_3} \tag{1}$$

where $D_3$ and $T_3$ are electric displacement and applied stress in direction ***3***, respectively. Under AC stress $T_3 = T_0 \sin(2\pi f t)$:

$$d_{33} = \frac{\partial D_3}{\partial (T_0 \sin(2\pi f t))} = \frac{1}{2T_0 \pi f \cos 2\pi f t} \frac{\partial D_3}{\partial t} = \frac{J_3}{2\pi f T_0 \cos 2\pi f t} \tag{2}$$

Thus, the obtained curve of current density $J_3 = 2\pi f d_{33} T_0 \cos(2\pi f t)$ shall be in an AC form with the same frequency as the stress and a magnitude ($J_0$) of $2\pi f d_{33} T_0$. Hence, the $d_{33}$ can be obtained from the measured current density:

$$d_{33} = J_0 / 2\pi f T_0 \tag{3}$$

**Supplementary Fig. 6a** show the measurements on PZT ceramic and PMN-0.3Pt crystal under 2MPa 1 Hz AC stress. The obtained $d_{33}$ of 440 pC/N for PZT and 1033 pC/N  for PMN-0.3PT are consistent with previous reports[4,5], which indicates the good reliability of our system.

**Supplementary Text 2 | Converse piezoelectricity measurements**

Here we first describe the shear strain measurement. As discussed in main text, the shear strain for both ferroelastic switch and $d_{35}$ measurements were investigated on single crystal samples with experiment setup in **Supplementary Fig. 5b**. Here, the ferroelastic strain is measured by bipolar electric field while $d_{35}$ was measure by unipolar electric field on pre-poled single crystals. The shear strain is defined by $S_5 = \alpha$ (**Supplementary Fig. 5b**). At small angle, $S_5$ can be obtained by $S_5 = \tan \alpha = d/T$, where $d$ and $T$ are shear displacement and sample thickness, respectively[6].

The *SE* curves of shear piezoelectric measurement show non-linearity (**Supplementary Fig. 5d**). This is attributed to ferroelastic switch contribution and the long time required for domain switching in OIHFs, which can be reflected by their low polarization reversal frequency (generally from 1-100 Hz)[7,8]. The linear region of a hysteretic *SE* curve is used to quantitatively calculate the piezoelectric coefficients[9,10]. Due to the hysteresis effect, the slope of **S**/**E** show electric field dependence. Therefore, $S_{max}/E_{max}$ is generally used to obtain so call 'large signal' piezoelectric coefficient to distinguish large and small **E** results[11-14]. Here, we use $S_5/E_3$ to estimate 'large signal' $d^*_{35}$.

Next, we use $LiH_3(SeO_3)_2$ as an example to simulate its top surface displacement under $E_3$ and show how $d_{33}$ is extracted from the displacement magnitude diagram of our OIHF sample. $LiH_3(SeO_3)_2$ is a piezoelectrics with the same *Cc* space group and hence piezoelectric tensor with the same symmetry as our OIHF. Assuming a $LiH_3(SeO_3)_2$ single crystal is placed as the setup in Fig. 2b, with [100], [010] and [001] crystal orientations as **X**, **Y** and **Z** axis, respectively, and the dimension of the crystal is $l_x = l_y = l_z = 1$mm. The initial geometry center of the top surface is the origin of the coordinates (**Supplementary Fig. 7a**). Under $E_3$, the piezoelectric and strain tensor of *Cc* space group piezoelectrics can be expressed as below[15]:

$$\begin{bmatrix} S_1 \\ S_2 \\ S_3 \\ S_4 \\ S_5 \\ S_6 \end{bmatrix} = \begin{bmatrix} d_{11} & 0 & d_{31} \\ d_{12} & 0 & d_{32} \\ d_{13} & 0 & d_{33} \\ 0 & d_{24} & 0 \\ d_{15} & 0 & d_{35} \\ 0 & d_{26} & 0 \end{bmatrix} \times \begin{bmatrix} 0 \\ 0 \\ E_3 \end{bmatrix} = \begin{bmatrix} d_{31} \times E_3 \\ d_{32} \times E_3 \\ d_{33} \times E_3 \\ 0 \\ d_{35} \times E_3 \\ 0 \end{bmatrix}$$

We first consider the case where $E_3$ and the ferroelectric polarization (projection along the **Z** axis) are in the same direction (**Supplementary Fig. 7b**). With 200 V voltage ($E_3 = 2$kV/cm$^2$) applied, the piezoelectric and strain tensor are[16]:

$$\begin{bmatrix} S_1 \\ S_2 \\ S_3 \\ S_4 \\ S_5 \\ S_6 \end{bmatrix} = \begin{bmatrix} 23.2 & 0 & -18.4 \\ -22.3 & 0 & 5.5 \\ -12.1 & 0 & 19.9 \\ 0 & -12.8 & 0 \\ -46.6 & 0 & 53.2 \\ 0 & -14.6 & 0 \end{bmatrix} \times \begin{bmatrix} 0 \\ 0 \\ 2 \end{bmatrix} = \begin{bmatrix} -3.68\,E^{-6} \\ 1.1\,E^{-6} \\ 3.98\,E^{-6} \\ 0 \\ 1.06E^{-5} \\ 0 \end{bmatrix}$$

The unit of $d_{ij}$ and $E_3$ are pm/V, kV/cm, respectively

Same as in our converse piezoelectric effect measurements, the $LiH_3(SeO_3)_2$ crystal was fixed by two thin probes on **XZ** surfaces. This quasi-free boundary condition allows the crystal to expand in *Z* direction and rotate freely in **XZ** plane (**Supplementary Fig. S7a**). Under this condition, the induced displacement by $S_5$ on points **X** = $x_a$ and -$x_a$ would have same magnitude but in opposite directions. The total displacement on *Z* direction is the sum of normal ($d_{33}$), transverse ($d_{31}$) and shear ($d_{35}$) piezoelectric contributions. Obviously, the normal piezoelectric contribution on top surface can be obtained by:

$$Z_{up1} = \frac{l_z \times S_3}{2} = \frac{l_z \times d_{33} \times E_3}{2} \tag{4}$$

Here $l_z \times S_3$ is divided by 2 since the bottom surface also undergoes expansion with the same magnitude. For transvers and shear piezoelectricity contribution, the displacement at a $x_a$ can be calculated as:

$$Z_{up2} = (x_a + D_{1a}) \times tan(S_5) = (x_a + \frac{d_{31} \times E_3 \times l_x}{2} \times \frac{x_a}{\frac{l_x}{2}}) \times tan(d_{35} \times E_3) \tag{5}$$

Here $D_{1a}$ is the deformation induced by $S_1$ ($d_{31}$) at $x_a$ position. The calculated $D_{1a}$ is far smaller compared with $x_a$. For example, even with $d_{31}$ = 10000 pm/V, the $D_{1a}$ is only $-2 \times 10^{-3} \times x_a$. This indicates $x_a$ show absolutely domination in $Z_{up2}$ and thus the contribution from $D_{1a}$ is negligible. Consequently, $Z_{up2}$ can be simplified as:

$$Z_{up2} = x_a \times tan(d_{35} \times E_3) = x_a \times d_{35} \times E_3 \tag{6}$$

with 1 × 1 × 1 mm dimension and $E_3$ = 2 kV/cm, the result of $Z_{up1}$, $Z_{up2}$ and the total displacement $Z_{up3} = Z_{up1} + Z_{up2}$ can be calculated as shown in **Supplementary Fig. 7b**. With the same method, the displacement under opposite electric field (-2 kV/cm) are also obtained as indicated in **Supplementary Fig. 7c**. The displacement magnitude under bipolar electric field can thus be calculated by $D = \frac{|Z_{up} - Z_{down}|}{2}$ (**Supplementary Fig. 7d)**. The displacement magnitude diagram of the top surface is illustrated in **Supplementary Fig. 7e**, where the data were recorded by the laser mapping facility on our OIHF samples (Fig. 2c). Obviously, the displacement at **X** = 0 line is not

affected by shear contribution. Consequently, for *Cc* space group piezoelectrics, $d_{33}$ can be extracted as:

$$d_{33} = \frac{S_{x=0}}{E_3} = \frac{2D_{x=0}}{V_3} \tag{7}$$

Here $V_3$ is the applied voltage. In our measurements, we use the average displacement at **X** = 0 line as the $D_{x=0}$. The displacement is multiplied by 2 in $d_{33}$ calculation since the strain is induced on both top and bottom surfaces with the same magnitude. Another noteworthy point is the shape of the displacement magnitude diagram. Because of the combination effects of normal and shear piezoelectric contributions, the displacement magnitude diagram shall have a valley shape with a zero-*D* line $x_0$ deviating from **X** = 0. As shown in **Supplementary Fig. 7b-d**, $x_0$ occurs where shear and normal piezoelectric contributions offset each other ($Z_1 = Z_2$), that is:

$$x_a \times d_{35} \times E_3 = \frac{l_z \times d_{33} \times E_3}{2} \tag{8}$$

The deviation between $x_0$ and **X** = 0 lines and the valley shape of displacement magnitude diagram reflect both shear and normal piezoelectric effects of piezoelectrics with a *Cc* space group. These two features can be used to verify the reliability of data. For example, for material with significant $d_{35}$ but negligible $d_{33}$, the $x_0$ position would appear nearly at **X** = 0. If other electric field induced effect contributes dominantly to the top surface displacement, the diagram of displacement magnitude will have a different shape.

**Supplementary Text 3 | Energy harvesting measurements**

Detail energy harvester measurements (along $d_{33}$ direction) were carried out to investigate the piezoelectric energy harvesting performance of this solid solution. The device is made by coating conductive sliver paste on single crystal wafer as the top and bottom electrodes. Copper wires was used to connect with the device and instruments. As indicated in **Supplementary Fig. 12a**, with x varying from 0 to 0.25, the voltage output shows monotonous increase from 90 to 210 V under compressive mechanical impact, which is consistent with the increase in $d_{33}$. Detail characterization was then performed on $x$ = 0.25 crystal wafer. **Supplementary Fig. 12b** and **c** show the loading dependent voltage and current outputs. As compressive force ranges from 10 to 50 N, the voltage and current density increase up to 210V and 63.8 μA/cm$^2$, respectively. To evaluate the maximum power density across load resistance, the output voltage and current were then measured across different resistance loading (**Supplementary Fig. 12d**). A maximum voltage output of 208 V is obtained across 100 MΩ, while a maximum current density of 65 μA/cm$^2$ is achieved with 100 Ω loading. Based on the maximum power transfer theorem, a maximum power output of 11 W/m$^2$ was obtained across a load impedance of 1 MΩ (Fig. 4d), which is two orders of magnitude those of traditional piezoelectric materials such as PMN-Pt and PVDF (Fig. 4f).

The energy conversion efficiency of $x$ = 0.25 crystal was estimated by:

$$\delta = \frac{E_{output}}{E_{input}} \times 100\% = \frac{\int_0^{t1} I^2 R dt}{\int_0^{l1} F dl} \times 100\% \quad (9)$$

where $I$, $R$, $t_1$, $F$, $l$, and $l_1$ are output current, loading resistance, produced pulse width, mechanical force, force exertion distance (deformation of the crystal in $d_{33}$ direction), and the largest force exertion distance, respectively. Here $l_1$ can be estimated by

$$l_1 = S \times H = \frac{T}{c_{33}} \times H \quad (10)$$

where $S$, $H$, $T$, $c_{33}$ are force induced strain, crystal thickness, stress and Young's modulus, respectively. According to above formulas and the resistance of 1 MΩ, stress of 3 MPa, $c_{33}$ of 800 MPa, the efficiency is calculated to be 4.06%.

The device was then integrated into capacitor to investigate the energy storage capability (**Supplementary Fig. 12e**). Under mechanical stress, the voltage for the 22-μF capacitor reaches 0.66V in 4 s, which corresponds to 14.2 μC charge and 5.39 μJ energy. The energy harvesting performance of this solid solution is compared with PZT and P(VDF-TrFE) under similar compressive pressure. As indicated in **Fig. 4c,** with 3 MPa loading, the voltage output of $x$ = 0.25 wafer (210 V) is one order of magnitude higher than that of PZT

(20.6 V) and PVDF (10 V). Considering its low weight density (2.12 g/cm$^3$, and PZT and PVDF are 7.8 and 1.8 g/cm$^3$, respectively), high voltage output, excellent transparence and mechanical softness, this hybrid ferroelectric show great promise as soft electronics especially for wearable sensor and energy harvester applications.

In order to evaluate the triboelectric contribution from the scotch tape to the voltage output of $x$ = 0.25 crystal, single electrode triboelectric measurement was conducted on the scotch tape. Here the tape was used as the triboelectric layer and the force was exerted by the metallic rod of the mechanical shaker (**Supplementary Fig. 14b**). As seen from Fig. Supplementary S14 c-d, the voltage output (<0.5 V) from the scotch tape is negligible compared to the piezoelectric signal of OIHF crystal. Next, we reversed the connection and repeat the measurement (**Supplementary Fig. 14e**). The voltage signal is completely reversed. This verifies the piezoelectric origin that the voltage signal is generated from the crystal instead from any artifices or other phenomena.

# Supplementary Figures

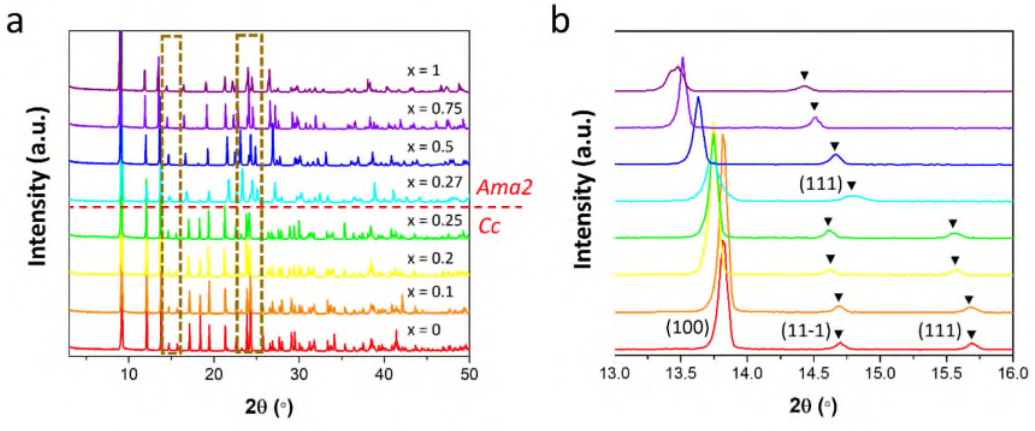


**Supplementary Figure 1 | Powder XRD patterns of this solid solution with various halide compositions.** (a) XRD data in the range from 3° to 50°. The red dash line indicates the phase transition between *Ama2* and *Cc*, and the dashed boxes indicate some obvious changes attributed to the *Cc*-*Ama*$_2$ transition. (b) Zoom-in of (100), (11-1) and (111) peaks region.

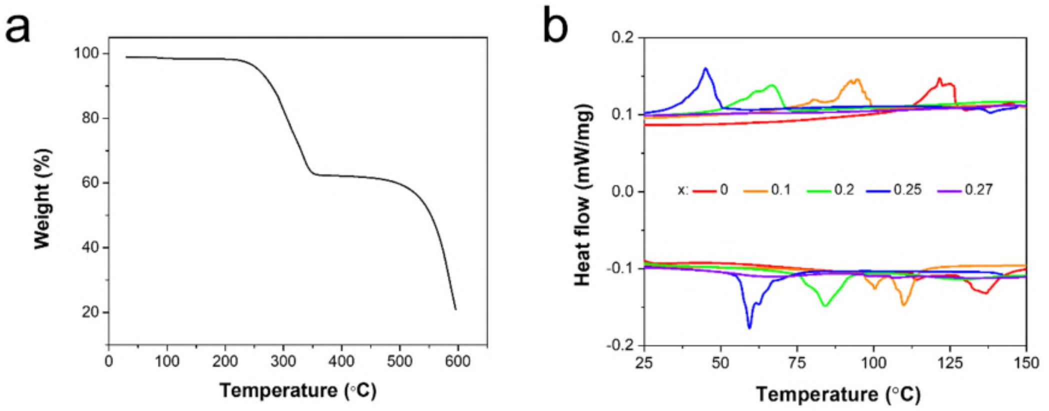


**Supplementary Figure 2 | TGA and DSC characterizations.** (**a**) TGA data of $C_6H_5N(CH_3)_3CdBr_2Cl_{0.75}I_{0.25}$ crystal, indicating good stability up to around 200 ℃. (**b**) DSC analysis of $C_6H_5N(CH_3)_3CdBr_2Cl_{1-x}I_x$ with selected compositions.

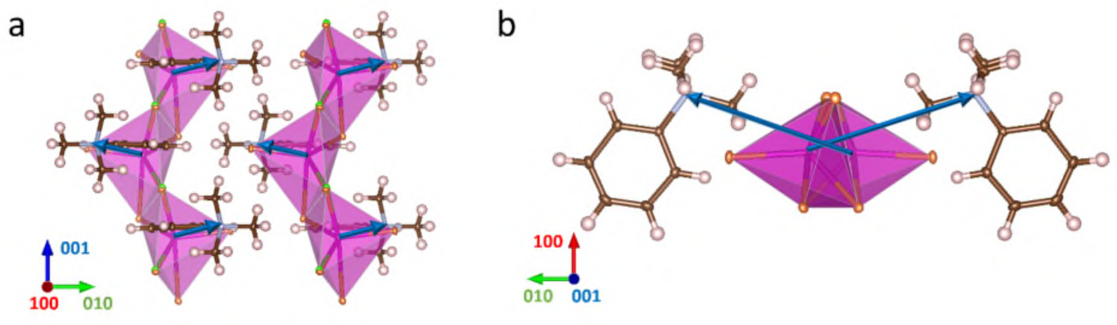


**Supplementary Figure 3 | Spontaneous polarization (blue arrow) in *x* = 0 crystal with perspective along [100] (a) and [001] directions (b).**

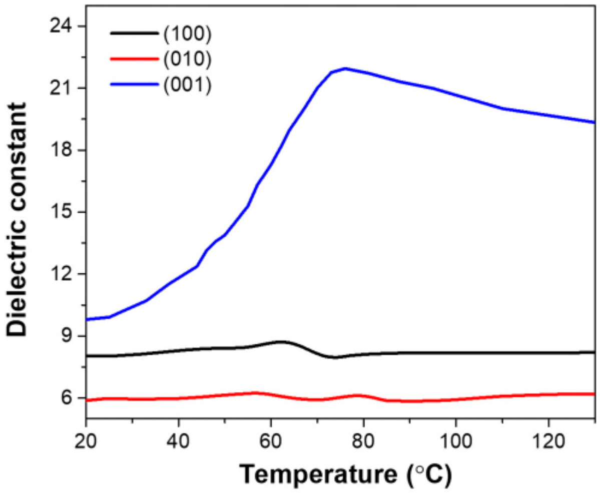


**Supplementary Figure 4 | Orientation dependent dielectric-temperature diagram of *x* = 0.25 crystal (10 kHz).** This dielectric behavior along the (001) direction, i.e., small change in dielectric constant near to transition temperature, is similar to those of classical ferroelectric systems (such as BTO and PIN-PMN-Pt) during the ferroelectric-ferroelectric phase transition[17,18].

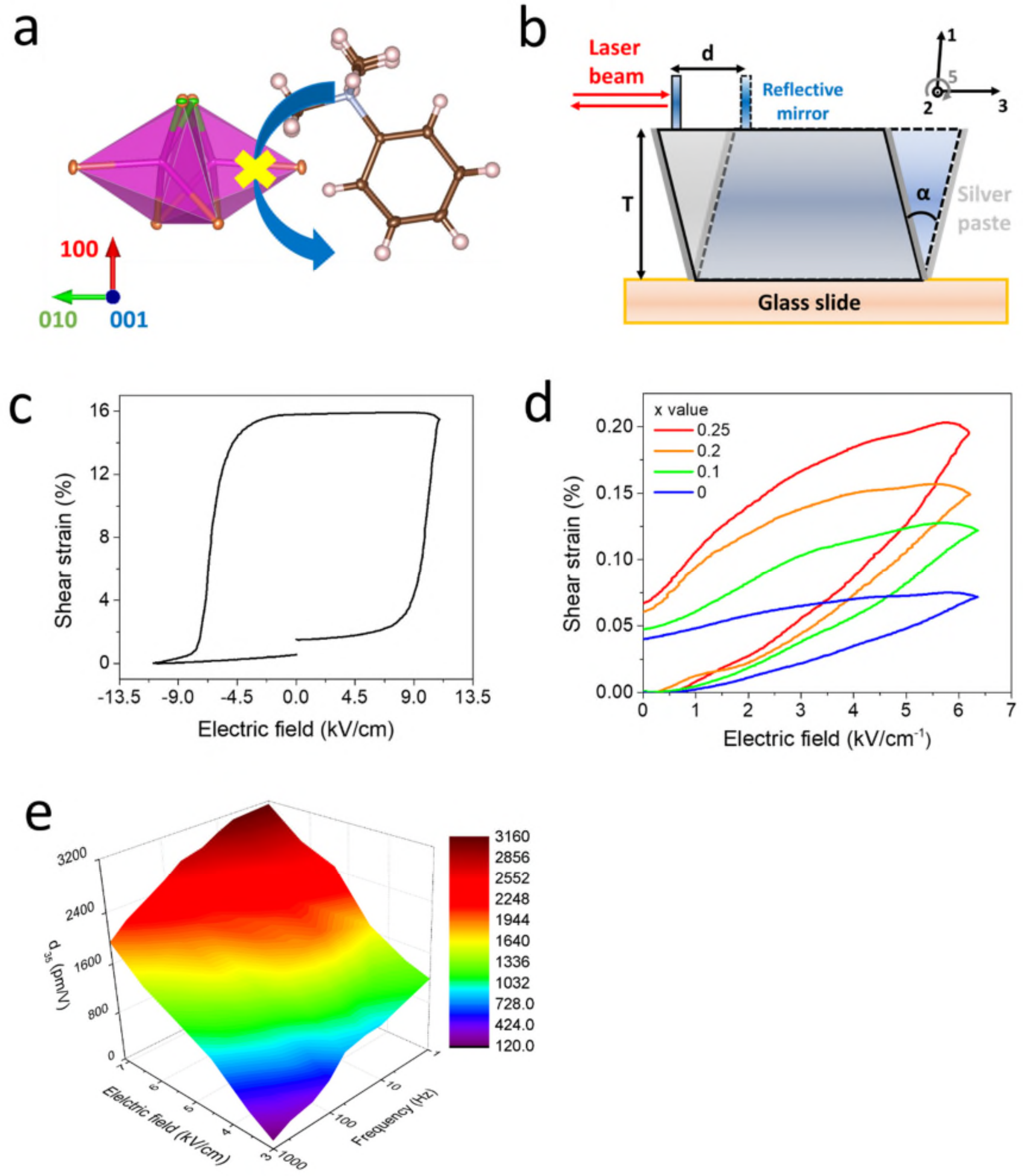


**Supplementary Figure 5 | Space-confinement effect, ferroelastic strain and shear piezoelectric ($d_{35}$) properties.** (a) The forbidden 180° polarization switching path. (b) Experiment setup for shear displacement measurement. The **1**, **2** and **3** directions correspond to crystallographic [100], [010] and [001] directions, respectively. (c) SE loop of $x$ = 0.25 crystal upon polarization switch with frequency of 1 Hz. (d) SE curve of selected compositions in large signal $d_{35}$ measurements. (e) **E** and $F$ dependent $d_{35}$ of $x$ = 0.25 crystal.

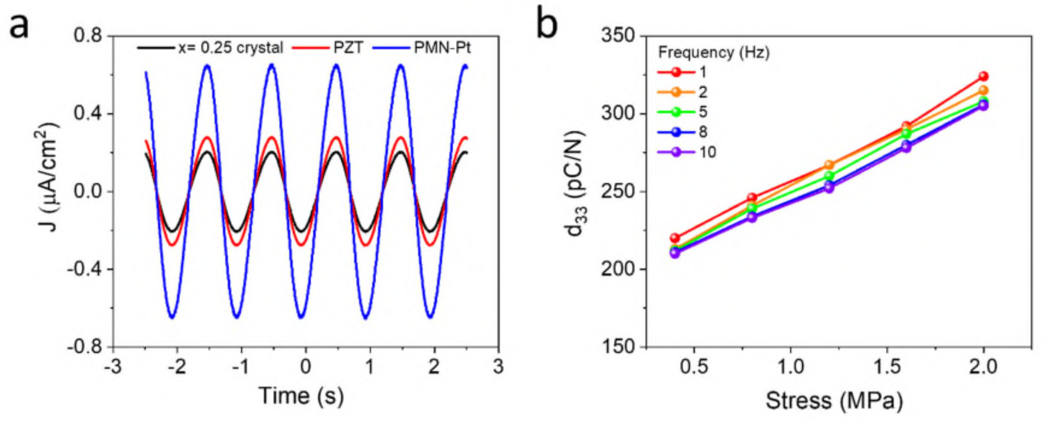


**Supplementary Figure 6 | Direction piezoelectric measurements.** (a) Generated current of $x$ = 0.25 crystal, PZT ceramics and PMN-0.3Pt crystals under 2MPa 1 Hz AC stress. (b) Stress and frequency dependence of $d_{33}$ of $x$ = 0.25 crystal, obtained by direct piezoelectric measurement (Supplementary Text 1).

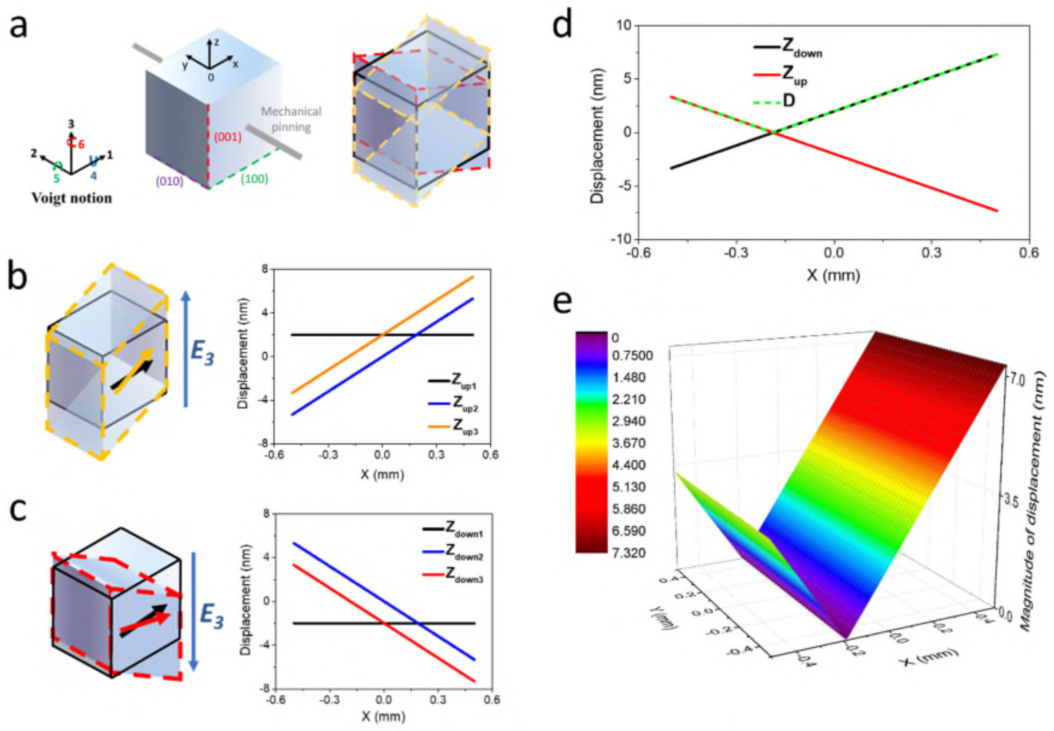


**Supplementary Figure 7 | Simulation of top-surface displacement magnitude diagram of $LiH_3(SeO_3)_2$.** (a) Left: Coordinate and crystal orientations of the simulated sample. Right: $S_5$ deformation under quasi-free boundary condition. (b) and (c) Crystal deformation (left) and $X$ dependent displacements (right) of the top surface under $E_3$ with the same ($Z_{up}$, b) and opposite directions ($Z_{down}$, c) to ferroelectric polarization (projection along $Z$). $Z_1$, and $Z_2$ are the displacements attributed to normal and shear piezoelectric effects. $Z_3 = Z_1 + Z_2$. (d) $X$ dependent displacements under positive and negative $E_3$ ($Z_{up}$ and $Z_{down}$, respectively) and magnitude of displacement ($D$) under bipolar $E_3$. (e) Simulated displacement magnitude diagram of crystal top surface under $E_3$ = 2 kV/cm.

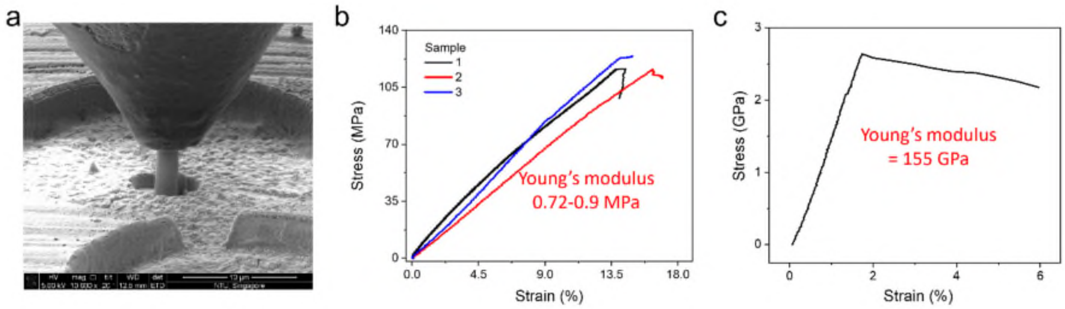


**Supplementary Figure 8 | Nano-indentation measurements.** (a) Scanning electron microscope image of a micrometer-pillar of $x = 0.25$ crystal compressed by tip. Strain-stress curves of three micro-pillar samples of $x = 0.25$ crystal (b) and standard Pt metal (c).

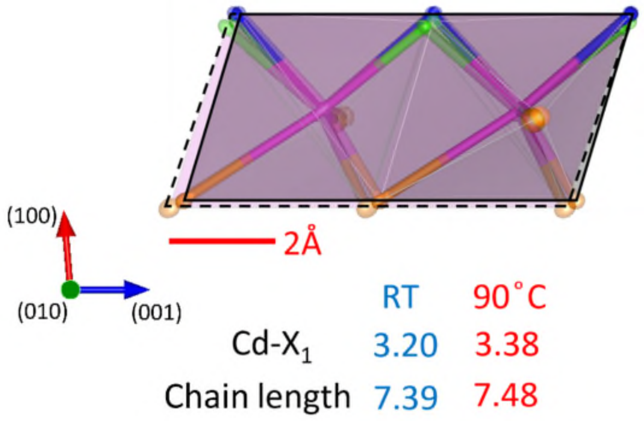


**Supplementary Figure 9 | Thermal expansion of metal-halide chain with *x* = 0.1 composition.** The solid and dash black line illustrate the profile of metal-halide polyhedron at room temperature (RT) and 90° C, respectively. The unit for Cd-X1 and chain length is Å.

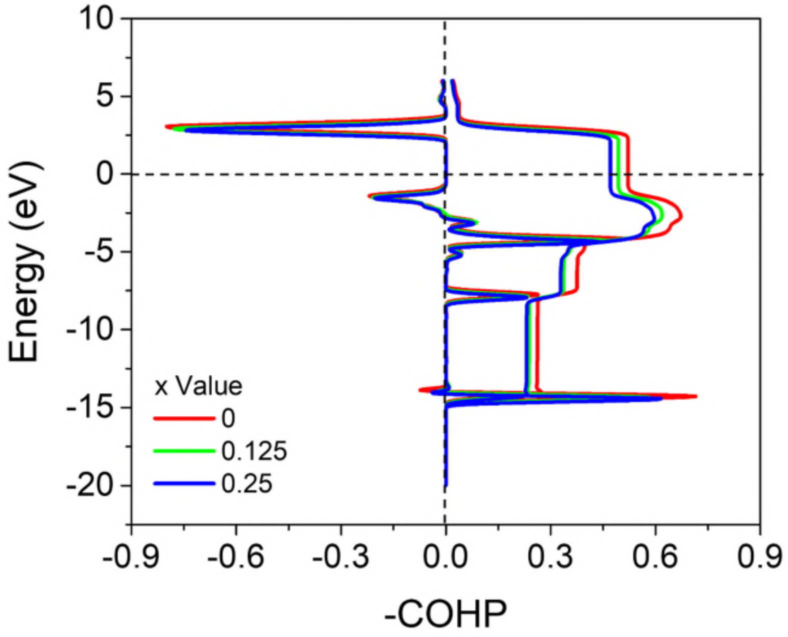


**Supplementary Figure 10 |** Calculated integrated crystal orbital Hamilton populations (COHP) of the Cd–X1 bond at selected compositions.

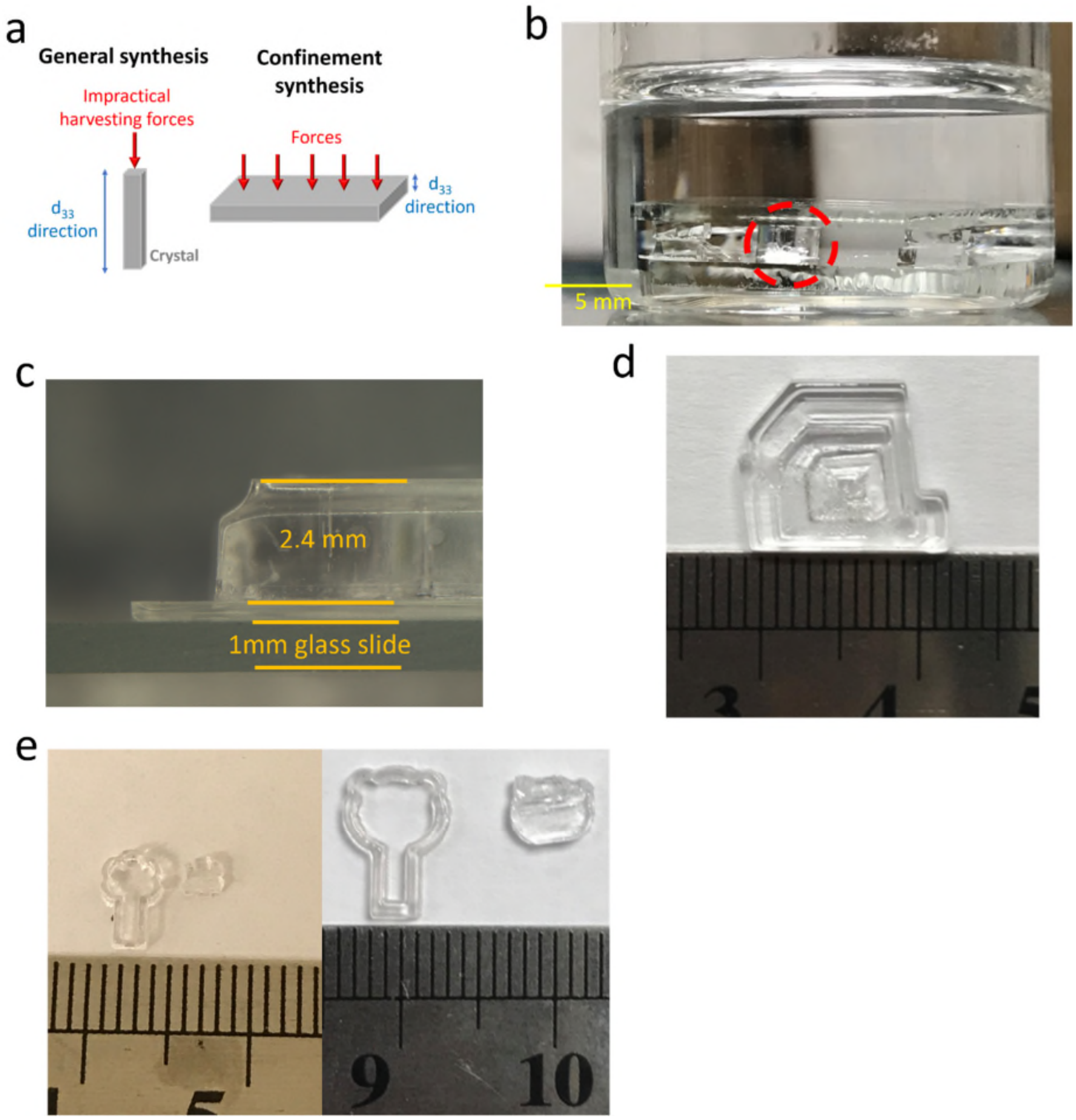


**Supplementary Figure 11 | Confinement synthesis of single crystal wafers.** (a) Schematic illustration of general and confinement synthesis. (b) Photo of confinement synthesis. Crystal is marked by red cycle. (c) Optical photo of a crystal wafer with thickness of 2.4 mm and (d) a crystal with width around 1 cm. (e) Customized-shape wafers (left) with corresponding plastic models (right).

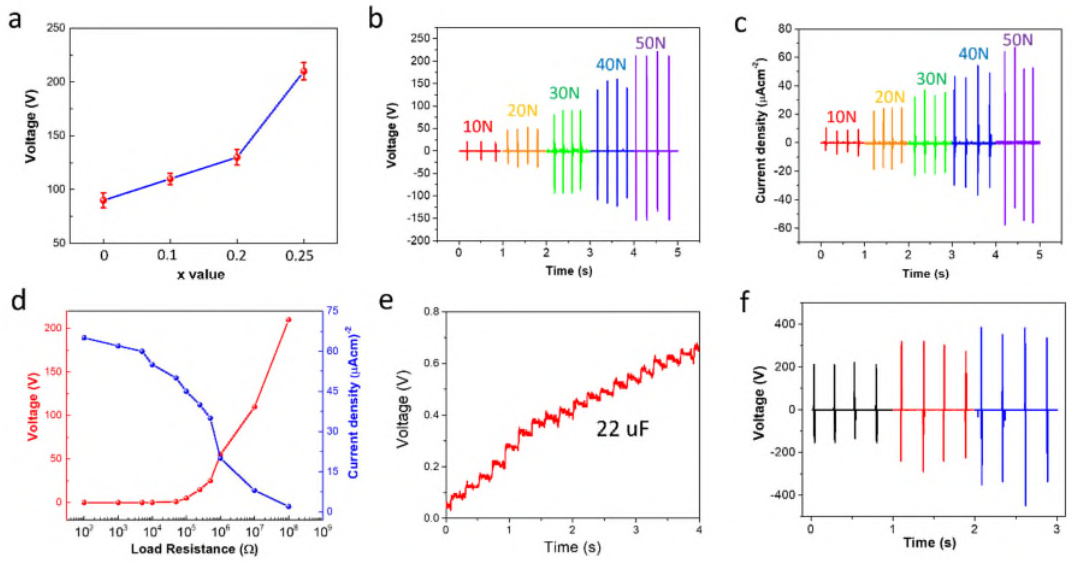


**Supplementary Figure 12 | Energy harvesting properties of $C_6H_5N(CH_3)_3CdBr_2Cl_{1-x}I_x$ crystal wafers.** (a) Voltage output of crystals with different halide compositions. The error bar indicates the voltage variation. Force dependent voltage (b) and current (c) outputs of $x$ = 0.25 crystal. (d) Voltage and current outputs of $x$ = 0.25 sample with different impedance loadings. (e) Traces of capacitor charging of $x$ = 0.25 crystal. (f) Voltage outputs of the devices made by connecting one, two and three $x$ = 0.25 crystals in series.

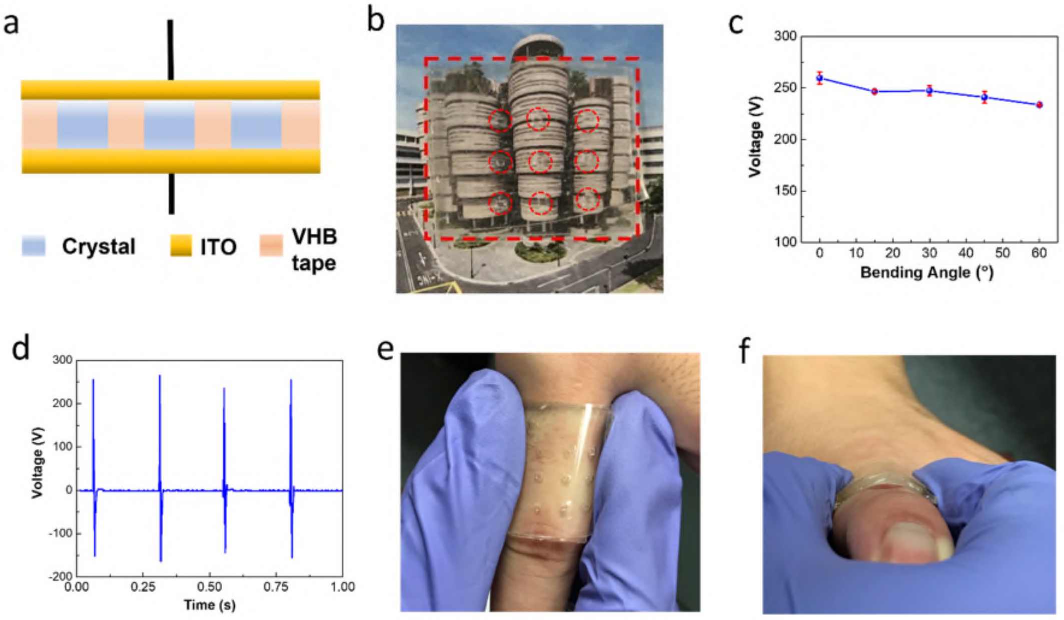


**Supplementary Figure 13 | Proof-of-concept flexible and transparent energy harvesting device based on $x$ = 0.25 crystals.** (a) Schematic of the device structure. (b) Photo of the device illustrating transparency. Thick dashed line and circles indicate the outline of the device and the crystals, respectively. (c) Voltage output under various bending angles. The error bar indicates the voltage variation. (d) Voltage output with zero bending. (e) and (f) photos of the flexible device on finger.

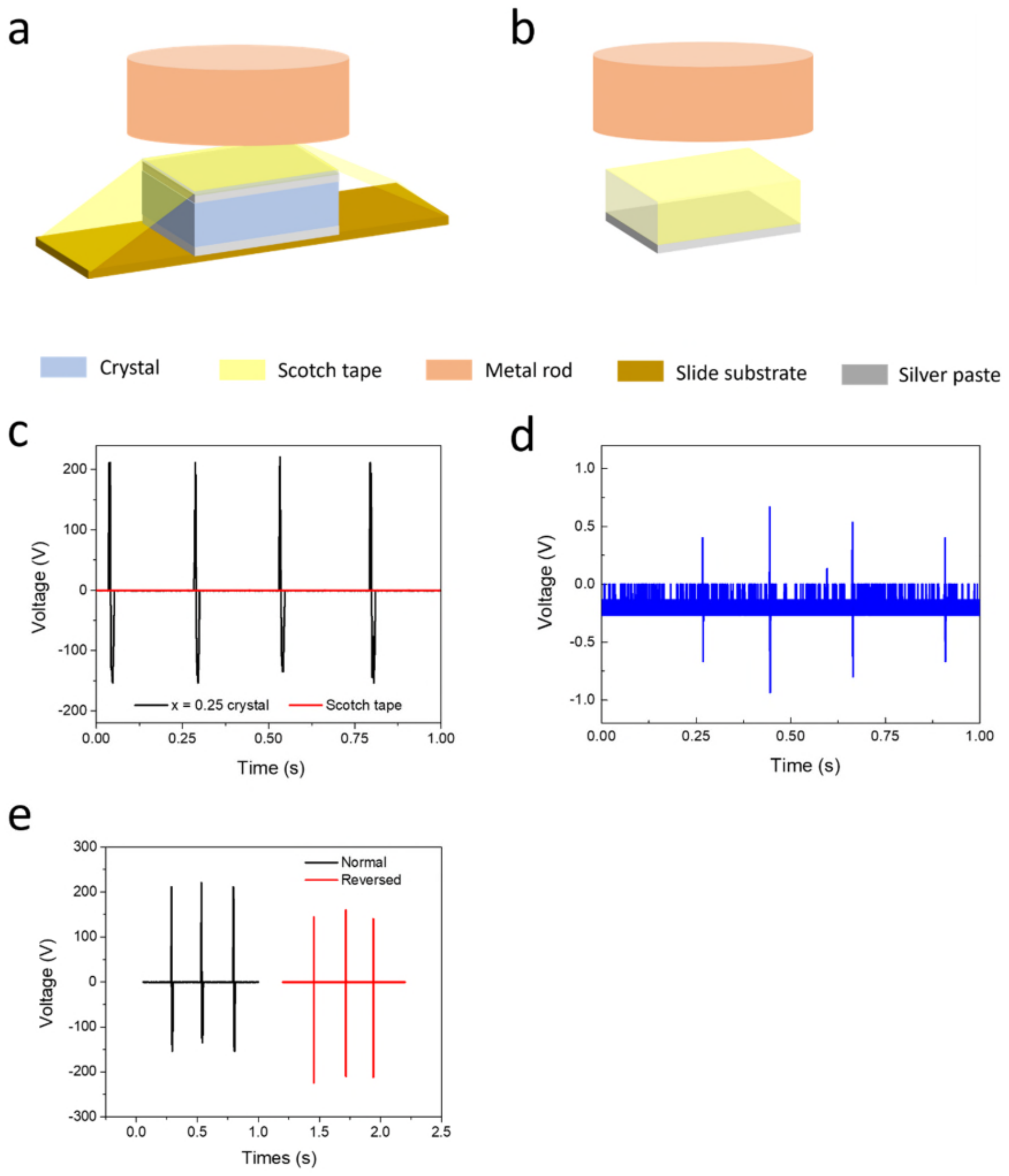


**Supplementary Figure 14 | Scotch tape triboelectric measurement and reversed electrical connection characterization.** Experiment setups for piezoelectric and triboelectric energy harvesting of $x$ = 0.25 crystal (a) and scotch tape (b). (c) Comparison between the voltage output of $x$ = 0.25 crystal (piezoelectric effect) and scotch tape (triboelectric effect). (d) Zoom-in view of the triboelectric signal from c. (e) Voltage output with normal and reversed electrical connection of $x$ = 0.25 crystal.

# Supplementary Tables

**Supplementary Table 1 | Crystallographic data of $C_6H_5N(CH_3)_3CdBr_2Cl_{(1-x)}I_x$ with different x value.**

| x | 0 | | 0.1 | | 0.25 | | 1 |
|---|---|---|---|---|---|---|---|
| Temperature (K) | 296 | 400 | 299 | 363 | 296 | 393 | 299 |
| Space group | *Cc* | *Ama2* | *Cc* | *Cc* | *Cc* | *Ama2* | *Ama2* |
| a (Å) | 12.955(5) | 12.802(10) | 12.9499(5) | 12.9880(6) | 12.9665(10) | 12.981(3) | 13.1561(4) |
| b (Å) | 14.714(6) | 14.611(11) | 14.7217(5) | 14.7337(6) | 14.7384(11) | 14.724(3) | 14.9090(6) |
| c (Å) | 7.385(3) | 7.559(5) | 7.3954(3) | 7.4847(3) | 7.4164(6) | 7.6681(13) | 7.8221(3) |
| β (°) | 95.094(7) | 90 | 95.096(2) | 94.336(2) | 94.971(2) | 90 | 90 |
| Volume ($Å^3$) | 1402.2(10) | 1413.9(18) | 1404.32(9) | 1428.18(11) | 1411.98(19) | 1465.6(5) | 1534.26(10) |
| Z | 4 | 4 | 4 | 4 | 4 | 4 | 4 |
| F (000) | 872.0 | 861.0 | 864.0 | 870 | 868 | 876 | 1056 |
| R factor (%) | 4.66 | 3.97 | 5.01 | 5.60 | 3.62 | 3.97 | 5.08 |
| Goodness-of-fit | 1.015 | 1.017 | 0.984 | 1.004 | 1.016 | 1.045 | 1.028 |

**Supplementary Table 2 | $d_{33}$, $g_{33}$ and FOM of different material systems**

| Material system | Compound | $d_{33}$ (pc/N) | $g_{33}$ ($10^{-3}$ VmN$^{-1}$) | FOM ($10^{-15}$m$^2$/N) | Reference |
|---|---|---|---|---|---|
| Hybrid ferroelectrics | $C_6H_5N(CH_3)_3CdBr_2Cl_{0.75}I_{0.25}$ | 324 (Direct) 367 pm/V (Converse) | 3595 | 1222855 | This work |
| | $(TMFM)_{0.26}(TMCM)_{0.74}CdCl_3$ | 1540 | 9506 | 14639240 | [19] |
| | $(ATHP)_2PbBr_4$ | 76 | 660 | 50160 | [20] |
| | $(R3HP)_2RbBiBr_6$ | 11 | 61 | 672 | [21] |
| | TMBM-$MnBr_3$ | 112 | 1120 | 125440 | [22] |
| | TMCM-$MnCl_3$ | 185 | 1681 | 310985 | [7] |
| Organic | PVDF | 33 | 310 | 10254 | [23] |
| | (β-CN)APB/ODPA | 16.5 | 533 | 8789 | [24] |
| | P(VDF-TrFE) | 25 | 237 | 5934 | [25] |
| | BOPVDF | 62 | 359 | 22274 | [26] |
| | $ImClO_4$ | 41 | 77 | 3157 | [27] |
| Inorganics | NBT-KBT | 150 | 20.5 | 3075 | [28] |
| | KNN-Li | 240 | 28.5 | 6840 | [29] |
| | $PbTiO_3$ | 143 | 129 | 19367 | [30] |
| | PMN-0.29Pt | 2000 | 40 | 80000 | [31] |
| | PZT-5A | 440 | 25 | 11000 | [31] |
| | BZT–50BCT | 546 | 15.23 | 8316 | [32] |
| | $BaTiO_3$ | 191 | 11.37 | 2172 | [32] |
| | PZT-4 | 258 | 32.39 | 8357 | [33] |
| | PIN-PMN-Pt | 1100 | 33.59 | 36949 | [33] |
| Composite materials | PZT/PVDF | 87.4 | 1097 | 95877 | [34] |
| | PZT/PDMS | 78 | 881 | 68718 | [35] |
| | KNNS-BNZH/Epoxy | 350 | 29.47 | 1031 | [36] |
| | PZT/SAC | 16 | 24 | 384 | [37] |
| | PZT/PVC | 15 | 43.18 | 645 | [38] |
| | PZT/Resin | 29 | 37 | 1073 | [39] |

| | | | | | |
|---|---|---|---|---|---|
| | PZT/PVDF-CTFE | 87 | 52 | 4524 | [40] |
| | NKLNT/Epoxy | 140 | 302 | 42280 | [41] |

As $g_{33}$ is defined under boundary condition of stress $\neq 0$ and strain = 0[42], we use $d_{33}$ obtained by direct piezoelectric effect (under 1Hz, 2 MPa stress) for Fig. 2f, ($d_{33}$ and $g_{33}$), Fig. 4f (*FOM*) and relevant calculations.

**Supplementary Table 3 | Young's modulus ($c_{33}$) of different materials**

| Material/Soft tissue | Young's modulus (GPa) | Reference |
|---|---|---|
| Ligament | 0.36 | [43] |
| Collagen | 0.5 | [44] |
| $C_6H_5N(CH_3)_3CdBr_2Cl_{0.75}I_{0.25}$ | 0.8 | This work |
| Human tendon | 1.2 | [45] |
| Human hair | 2 | [46] |
| PVDF | 2.2 | [47] |
| Nylon | 2.7 | [48] |
| $CH_3NH_3PbI_3$ | 12.5 | [49] |
| PZT | 74 | [50] |
| Stain less steel | 230 | [51] |

**Supplementary Table 4 | Power density and softness of different materials**

| Material system | Compound | Elastic modulus (GPa) | Power density (μW/cm$^2$) | Reference |
|---|---|---|---|---|
| Hybrid ferroelectrics | $(PTMA)CdBr_2Cl_{0.75}I_{0.25}$ | 0.8 | 1100 | This work |
| | PMO-NiNCS | 7.5* | 50.26 | [52] |
| | $(ATHP)_2PbBr_2Cl_2$ | 8* | 1.7 | [53] |
| | $[BnNMe_2P]CdBr_4$ | 8* | 37.1 | [54] |
| | $[Ph3MeP]2[CuCl_4]$ | 15* | 14.1 | [55] |
| Inorganics | PMN-Pt | 75 | 58.82 | [56,57] |
| | PZT | 74 | 5.92 | [50,58] |
| | BCZT | 118 | 450 | [59,60] |
| | PIN-PMN-Pt | 132 | 21.875 | [61,62] |
| | $BaTiO_3$ | 67 | 12 | [63,64] |
| | PIN-PMN-PT arrays | 132 | 61.25 | [65] |
| | BNT-BKT | 136 | 1.381 | [66,67] |
| Organics | P(VDF-TrFE) | 1.5 | 4.63 | [68,69] |
| | PVDF | 2.2 | 3.325 | [47,70] |
| | Collagen | 0.5 | 0.16 | [44,71] |
| | Bilayer P(VDF-TrFE) | 1.5 | 1.77 | [72] |

To make comparison of the harvesting performance among different piezoelectrics, here we choose devices based on single piezoelectric component (For example, composites like PVDF/PZT are not included here).

*The elastic modulus of most organic-inorganic hybrids are in the range of 5-70 GPa[73]. Due to the lack of Young's moduli data of OIHF crystals, the Young's moduli of these materials are taken from other OIHF systems with similar dimension or compositions[73].

## Supplementary reference


1 Berlincourt, D. & Krueger, H. H. A. Domain Processes in Lead Titanate Zirconate and Barium Titanate Ceramics. *J. Appl. Phys.* **30**, 1804-1810, doi:10.1063/1.1735059 (1959).

2 Stewart, M. & Cain, M. in *Characterisation of Ferroelectric Bulk Materials and Thin Films* (Springer Dordrecht, Netherlands, 2014).

3 Yang, M.-M. *et al.* Piezoelectric and pyroelectric effects induced by interface polar symmetry. *Nature* **584**, 377-381, doi:10.1038/s41586-020-2602-4 (2020).

4 Li, F., Xu, Z., Wei, X. & Yao, X. Determination of temperature dependence of piezoelectric coefficients matrix of lead zirconate titanate ceramics by quasi-static and resonance method. *J. Phys. D: Appl. Phys.* **42**, 095417, doi:10.1088/0022-3727/42/9/095417 (2009).

5 Shrout, T. R., Chang, Z. P., Kim, N. & Markgraf, S. Dielectric behavior of single crystals near the (1−X) Pb(Mg1/3Nb2/3)O3-(x) PbTiO3 morphotropic phase boundary. *Ferroelectrics Letters Section* **12**, 63-69, doi:10.1080/07315179008201118 (1990).

6 Orgéas, L. & Favier, D. Stress-induced martensitic transformation of a NiTi alloy in isothermal shear, tension and compression. *Acta Mater.* **46**, 5579-5591, doi:https://doi.org/10.1016/S1359-6454(98)00167-0 (1998).

7 You, Y. M., Liao, W. Q., Zhao, D., Ye, H. Y., Zhang, Y., Zhou, Q. An organic-inorganic perovskite ferroelectric with large piezoelectric response. *Science* **357**, 306-309 (2017).

8 Zhang, Y. *et al.* Highly Efficient Red-Light Emission in An Organic–Inorganic Hybrid Ferroelectric: (Pyrrolidinium)MnCl3. *J. Am. Chem. Soc.* **137**, 4928-4931, doi:10.1021/jacs.5b01680 (2015).

9 Park, S.-E. & Shrout, T. R. Ultrahigh strain and piezoelectric behavior in relaxor based ferroelectric single crystals. *J. Appl. Phys.* **82**, 1804-1811, doi:10.1063/1.365983 (1997).

10 Liu, W. & Ren, X. Large Piezoelectric Effect in Pb-Free Ceramics. *Phys. Rev. Lett.* **103**, 257602, doi:10.1103/PhysRevLett.103.257602 (2009).

11 Viehland, D. *et al.* Effect of uniaxial stress on the large-signal electromechanical properties of electrostrictive and piezoelectric lead magnesium niobate lead titanate ceramics. *J. Appl. Phys.* **95**, 1969-1972, doi:10.1063/1.1641960 (2004).

12 Zhang, S.-T., Kounga, A. B., Aulbach, E., Ehrenberg, H. & Rödel, J. Giant strain in lead-free piezoceramics Bi0.5Na0.5TiO3–BaTiO3–K0.5Na0.5NbO3 system. *Appl. Phys. Lett.* **91**, 112906, doi:10.1063/1.2783200 (2007).

13 Kerkamm, I., Hiller, P., Granzow, T. & Rödel, J. Correlation of small- and large-signal properties of lead zirconate titanate multilayer actuators. *Acta Mater.* **57**, 77-86, doi:https://doi.org/10.1016/j.actamat.2008.08.057 (2009).

14 Hao, J., Li, W., Zhai, J. & Chen, H. Progress in high-strain perovskite piezoelectric ceramics. *Materials Science and Engineering: R: Reports* **135**, 1-57, doi:https://doi.org/10.1016/j.mser.2018.08.001 (2019).

15 IEEE Standard on Piezoelectricity. *ANSI/IEEE Std 176-1987*, 0_1, doi:10.1109/IEEESTD.1988.79638 (1988).

16 Berlincourt, D., Cook, W. R., Jnr & Rander, M. E. Piezoelectric, dielectric, and pyroelectric constants of LiH3(SeO3)2. *Acta Crystallogr.* **16**, 163-165, doi:doi:10.1107/S0365110X63000426 (1963).

17 Zhang, S. *et al.* Electromechanical characterization of Pb(In0.5Nb0.5)O3–Pb(Mg1/3Nb2/3)O3–PbTiO3 crystals as a function of crystallographic orientation and temperature. *J. Appl. Phys.* **105**, 104506, doi:10.1063/1.3131622 (2009).

18 Merz, W. J. The Electric and Optical Behavior of BaTiO3 Single-Domain Crystals. *Phys. Rev.* **76**, 1221-1225, doi:10.1103/PhysRev.76.1221 (1949).

19 Liao, W.-Q. *et al.* A molecular perovskite solid solution with piezoelectricity stronger than lead zirconate titanate. *Science* **363**, 1206-1210, doi:10.1126/science.aav3057 (2019).

20 Chen, X.-G. *et al.* Two-Dimensional Layered Perovskite Ferroelectric with Giant Piezoelectric Voltage Coefficient. *J. Am. Chem. Soc.* **142**, 1077-1082, doi:10.1021/jacs.9b12368 (2020).

21 He, L. *et al.* Coexisting Ferroelectric and Ferroelastic Orders in Rare 3D Homochiral Hybrid Bimetal Halides. *Chem. Mater.* **33**, 6233-6239, doi:10.1021/acs.chemmater.1c02084 (2021).

22 Liao, W.-Q., Tang, Y.-Y., Li, P.-F., You, Y.-M. & Xiong, R.-G. Large Piezoelectric Effect in a Lead-Free Molecular Ferroelectric Thin Film. *J. Am. Chem. Soc.* **139**, 18071-18077, doi:10.1021/jacs.7b10449 (2017).

23 Xu, R. & Kim, S. Figures of merits of piezoelectric materials in energy harvesters. *Proceedings of the PowerMEMS*, 464-467 (2012).

24 Park, C., Ounaies, Z., Wise, K. E. & Harrison, J. S. In situ poling and imidization of amorphous piezoelectric polyimides. *Polymer* **45**, 5417-5425, doi:10.1016/j.polymer.2004.05.057 (2004).

25 Sun, Q. *et al.* The Dependence of Acoustic Emission Performance on the Crystal Structures, Dielectric, Ferroelectric, and Piezoelectric Properties of the P(VDF-TrFE) Sensors. *IEEE Transactions on Ultrasonics, Ferroelectrics, and Frequency Control* **67**, 975-983, doi:10.1109/TUFFC.2019.2959353 (2020).

26 Huang, Y. *et al.* Enhanced piezoelectricity from highly polarizable oriented amorphous fractions in biaxially oriented poly(vinylidene fluoride) with pure β crystals. *Nat. Commun.* **12**, 675, doi:10.1038/s41467-020-20662-7 (2021).

27 Zhang, Y. *et al.* A Molecular Ferroelectric Thin Film of Imidazolium Perchlorate That Shows Superior Electromechanical Coupling. *Angew. Chem. Int. Ed.* **53**, 5064-5068, doi:10.1002/anie.201400348 (2014).

28 Zhao, S., Li, G., Ding, A., Wang, T. & Yin, Q. Ferroelectric and piezoelectric properties of (Na, K)0.5Bi0.5TiO3lead free ceramics. *J. Phys. D: Appl. Phys.* **39**, 2277-2281, doi:10.1088/0022-3727/39/10/042 (2006).

29 Hollenstein, E., Davis, M., Damjanovic, D. & Setter, N. Piezoelectric properties of Li- and Ta-modified (K0.5Na0.5)NbO3 ceramics. *Appl. Phys. Lett.* **87**, 182905, doi:10.1063/1.2123387 (2005).

30 Yan, Y., Zhou, J. E., Maurya, D., Wang, Y. U. & Priya, S. Giant piezoelectric voltage coefficient in grain-oriented modified PbTiO3 material. *Nat. Commun.* **7**, 13089, doi:10.1038/ncomms13089 (2016).

31 Zhang, Y. *et al.* Fabrication of angle beam two-element ultrasonic transducers with PMN–PT single crystal and PMN–PT/epoxy 1–3 composite for NDE applications. *Sensors and Actuators A: Physical* **168**, 223-228, doi:https://doi.org/10.1016/j.sna.2011.04.004 (2011).

32 Gao, J., Xue, D., Liu, W., Zhou, C. & Ren, X. in *Actuators.* 24 (Multidisciplinary Digital Publishing Institute).

33 Li, G. *et al.* Investigation of High-Power Properties of PIN-PMN-PT Relaxor-Based Ferroelectric Single Crystals and PZT-4 Piezoelectric Ceramics. *IEEE Transactions on Ultrasonics, Ferroelectrics, and Frequency Control* **67**, 1641-1646, doi:10.1109/TUFFC.2020.2979217 (2020).

34 Chang, J. *et al.* Large d33 and enhanced ferroelectric/dielectric properties of poly(vinylidene fluoride)-based composites filled with Pb(Zr0.52Ti0.48)O3 nanofibers. *RSC Advances* **5**, 51302-51307, doi:10.1039/C5RA07932B (2015).

35 Sappati, K. K. & Bhadra, S. Flexible Piezoelectric 0–3 PZT-PDMS Thin Film for Tactile Sensing. *IEEE Sens. J.* **20**, 4610-4617, doi:10.1109/JSEN.2020.2965083 (2020).

36 Ke, Q., Liew, W. H., Tao, H., Wu, J. & Yao, K. KNNS-BNZH Lead-Free 1–3 Piezoelectric Composite for Ultrasonic and Photoacoustic Imaging. *IEEE Transactions on Ultrasonics, Ferroelectrics, and Frequency Control* **66**, 1395-1401, doi:10.1109/TUFFC.2019.2914464 (2019).

37 Huang, S. *et al.* Piezoelectric properties of 0-3 PZT/sulfoaluminate cement composites. *Smart Mater. Struct.* **13**, 270-274, doi:10.1088/0964-1726/13/2/004 (2004).

38 Liu, X. F. *et al.* in *2009 18th IEEE International Symposium on the Applications of Ferroelectrics.* 1-4.

39 Nhuapeng, W. & Tunkasiri, T. Properties of 0–3 Lead Zirconate Titanate–Polymer Composites Prepared in a Centrifuge. *J. Am. Ceram. Soc.* **85**, 700-702, doi:https://doi.org/10.1111/j.1151-2916.2002.tb00154.x (2002).

40 Choi, Y. J. *et al.* Dielectric and piezoelectric properties of ceramic-polymer composites with 0–3 connectivity type. *J. Electroceram.* **30**, 30-35, doi:10.1007/s10832-012-9706-7 (2013).

41 Shen, Z.-Y., Li, J.-F., Chen, R., Zhou, Q. & Shung, K. K. Microscale 1-3-Type (Na,K)NbO3-Based Pb-Free Piezocomposites for High-Frequency Ultrasonic Transducer Applications. *J.*

*Am. Ceram. Soc.* **94**, 1346-1349, doi:https://doi.org/10.1111/j.1551-2916.2011.04508.x (2011).

42 Uchino, K. in *Advanced Piezoelectric Materials (Second Edition)* (ed Kenji Uchino) 1-92 (Woodhead Publishing, 2017).

43 Lai, Y.-S. *et al.* The Effect of Graft Strength on Knee Laxity and Graft In-Situ Forces after Posterior Cruciate Ligament Reconstruction. *PLOS ONE* **10**, e0127293, doi:10.1371/journal.pone.0127293 (2015).

44 van der Rijt, J. A. J., van der Werf, K. O., Bennink, M. L., Dijkstra, P. J. & Feijen, J. Micromechanical Testing of Individual Collagen Fibrils. *Macromolecular Bioscience* **6**, 697-702, doi:https://doi.org/10.1002/mabi.200600063 (2006).

45 Maganaris, C. N. & Paul, J. P. In vivo human tendon mechanical properties. *The Journal of Physiology* **521**, 307-313, doi:https://doi.org/10.1111/j.1469-7793.1999.00307.x (1999).

46 Hu, Z. *et al. Measurement of Young's modulus and Poisson's ratio of human hair using optical techniques*. Vol. 7522 EM (SPIE, 2010).

47 Sengupta, D. *et al.* Characterization of single polyvinylidene fluoride (PVDF) nanofiber for flow sensing applications. *AIP Advances* **7**, 105205, doi:10.1063/1.4994968 (2017).

48 Perkins, W. G. & Porter, R. S. Solid-state extrusion of nylons 11 and 12: processing, morphology and properties. *Journal of Materials Science* **16**, 1458-1470, doi:10.1007/BF02396864 (1981).

49 Lim, K.-G., Han, T.-H. & Lee, T.-W. Engineering electrodes and metal halide perovskite materials for flexible/stretchable perovskite solar cells and light-emitting diodes. *Energ. Environ. Sci* **14**, 2009-2035, doi:10.1039/D0EE02996C (2021).

50 Ewart, L. M., Mclaughlin, E. A., Robinson, H. C., Stace, J. J. & Amin, A. Mechanical and electromechanical properties of pmnt single crystals for naval sonar transducers. *IEEE Transactions on Ultrasonics, Ferroelectrics, and Frequency Control* **54**, 2469-2473, doi:10.1109/TUFFC.2007.561 (2007).

51 Tromas, C., Stinville, J. C., Templier, C. & Villechaise, P. Hardness and elastic modulus gradients in plasma-nitrided 316L polycrystalline stainless steel investigated by nanoindentation tomography. *Acta Mater.* **60**, 1965-1973, doi:https://doi.org/10.1016/j.actamat.2011.12.012 (2012).

52 Vijayakanth, T., Ram, F., Praveenkumar, B., Shanmuganathan, K. & Boomishankar, R. Piezoelectric Energy Harvesting from a Ferroelectric Hybrid Salt [Ph3MeP]4[Ni(NCS)6] Embedded in a Polymer Matrix. *Angew. Chem. Int. Ed.* **n/a**, doi:10.1002/anie.202001250.

53 Khan, A. A. *et al.* Superior transverse piezoelectricity in organic-inorganic hybrid perovskite nanorods for mechanical energy harvesting. *Nano Energy* **86**, 106039, doi:https://doi.org/10.1016/j.nanoen.2021.106039 (2021).

54 Deswal, S. *et al.* Flexible Composite Energy Harvesters from Ferroelectric A2MX4-Type Hybrid Halogenometallates. *Chem. Mater.* **31**, 4545-4552, doi:10.1021/acs.chemmater.9b01227 (2019).

55 Sahoo, S. *et al.* Ferroelectricity and Piezoelectric Energy Harvesting of Hybrid A2BX4-Type Halogenocuprates Stabilized by Phosphonium Cations. *ACS Materials Au* **2**, 124-131, doi:10.1021/acsmaterialsau.1c00046 (2022).

56 Hwang, G.-T. *et al.* Self-Powered Cardiac Pacemaker Enabled by Flexible Single Crystalline PMN-PT Piezoelectric Energy Harvester. *Adv. Mater.* **26**, 4880-4887, doi:https://doi.org/10.1002/adma.201400562 (2014).

57 Cordero, F. Elastic Properties and Enhanced Piezoelectric Response at Morphotropic Phase Boundaries. *Materials* **8**, 8195-8245 (2015).

58 Jin, W. *et al.* High-performance piezoelectric energy harvesting of vertically aligned Pb (Zr, Ti) O 3 nanorod arrays. *RSC advances* **8**, 7422-7427 (2018).

59 Coondoo, I. *et al.* A comparative study of structural and electrical properties in lead-free BCZT ceramics: Influence of the synthesis method. *Acta Mater.* **155**, 331-342, doi:https://doi.org/10.1016/j.actamat.2018.05.029 (2018).

60 Liu, S. *et al.* A flexible and lead-free BCZT thin film nanogenerator for biocompatible energy harvesting. *Materials Chemistry Frontiers* **5**, 4682-4689, doi:10.1039/D1QM00145K (2021).

61 Zeng, Z. *et al.* A flexible, sandwich structure piezoelectric energy harvester using PIN-PMN-PT/epoxy 2-2 composite flake for wearable application. *Sensors and Actuators A: Physical* **265**,

62-69, doi:https://doi.org/10.1016/j.sna.2017.07.059 (2017).
62 Zhang, W. *et al.* Determination of the Mechanical Properties of PIN–PMN–PT Bulk Single Crystals by Nanoindentation. *Crystals* **10**, 28 (2020).
63 Yan, J. & Jeong, Y. G. High Performance Flexible Piezoelectric Nanogenerators based on BaTiO3 Nanofibers in Different Alignment Modes. *ACS Appl. Mater. Interfaces* **8**, 15700-15709, doi:10.1021/acsami.6b02177 (2016).
64 Lim, J. *et al.* All-inkjet-printed flexible piezoelectric generator made of solvent evaporation assisted BaTiO3 hybrid material. *Nano Energy* **41**, 337-343, doi:https://doi.org/10.1016/j.nanoen.2017.09.046 (2017).
65 Zeng, Z. *et al.* High performance of macro-flexible piezoelectric energy harvester using a 0.3PIN-0.4Pb(Mg1/3Nb2/3)O3-0.3PbTiO3flake array. *Smart Mater. Struct.* **25**, 125015, doi:10.1088/0964-1726/25/12/125015 (2016).
66 Maria Joseph Raj, N. P., Khandelwal, G. & Kim, S.-J. 0.8BNT–0.2BKT ferroelectric-based multimode energy harvester for self-powered body motion sensors. *Nano Energy* **83**, 105848, doi:https://doi.org/10.1016/j.nanoen.2021.105848 (2021).
67 Kim, C.-Y., Sekino, T. & Niihara, K. Optical, mechanical, and dielectric properties of Bi1/2Na1/2TiO3 thin film synthesized by sol–gel method. *J. Sol-Gel Sci. Technol.* **55**, 306-310, doi:10.1007/s10971-010-2250-5 (2010).
68 Choi, Y.-Y. *et al.* Vertically aligned P(VDF-TrFE) core-shell structures on flexible pillar arrays. *Scientific Reports* **5**, 10728, doi:10.1038/srep10728 (2015).
69 Kim, S. *et al.* P(VDF-TrFE) Film on PDMS Substrate for Energy Harvesting Applications. *Applied Sciences* **8**, 213 (2018).
70 Yu, Y. *et al.* Biocompatibility and in vivo operation of implantable mesoporous PVDF-based nanogenerators. *Nano Energy* **27**, 275-281, doi:https://doi.org/10.1016/j.nanoen.2016.07.015 (2016).
71 Vivekananthan, V. *et al.* Biocompatible Collagen Nanofibrils: An Approach for Sustainable Energy Harvesting and Battery-Free Humidity Sensor Applications. *ACS Appl. Mater. Interfaces* **10**, 18650-18656, doi:10.1021/acsami.8b02915 (2018).
72 Bhavanasi, V., Kumar, V., Parida, K., Wang, J. & Lee, P. S. Enhanced Piezoelectric Energy Harvesting Performance of Flexible PVDF-TrFE Bilayer Films with Graphene Oxide. *ACS Appl. Mater. Interfaces* **8**, 521-529, doi:10.1021/acsami.5b09502 (2016).
73 Ji, L.-J., Sun, S.-J., Qin, Y., Li, K. & Li, W. Mechanical properties of hybrid organic-inorganic perovskites. *Coord. Chem. Rev.* **391**, 15-29, doi:https://doi.org/10.1016/j.ccr.2019.03.020 (2019).
