# Supplementary material for: Bond engineering of molecular ferroelectrics renders soft and high-performance piezoelectric energy harvesting materials
Source: Nat Commun. 2022 Sep 24;13:5607. doi: 10.1038/s41467-022-33325-6 (PMC9509372; doi:10.1038/s41467-022-33325-6)
Supplement: Supplementary file 3 — Lasing Reporting Summary [file 41467_2022_33325_MOESM3_ESM.pdf]

nature research

Corresponding author(s): Yuzhong Hu
Hong Jin Fan
Pooi See Lee

Lasing Reporting Summary

Nature Research wishes to improve the reproducibility of the work that we publish. This form is intended for publication with all accepted papers reporting claims of lasing and provides structure for consistency and transparency in reporting. Some list items might not apply to an individual manuscript, but all fields must be completed for clarity.

For further information on Nature Research policies, including our data availability policy, see Authors & Referees.

ü Experimental design

Please check: are the following details reported in the manuscript?

1. Threshold

Plots of device output power versus pump power over a wide range of values indicating a clear threshold

☐ Yes
☒ No

We have not done any laser measurement in our work.

2. Linewidth narrowing

Plots of spectral power density for the emission at pump powers below, around, and above the lasing threshold, indicating a clear linewidth narrowing at threshold

☐ Yes
☒ No

No laser measurement involved

Resolution of the spectrometer used to make spectral measurements

☐ Yes
☒ No

No laser measurement involved

3. Coherent emission

Measurements of the coherence and/or polarization of the emission

☐ Yes
☒ No

No laser measurement involved

4. Beam spatial profile

Image and/or measurement of the spatial shape and profile of the emission, showing a well-defined beam above threshold

☐ Yes
☒ No

No laser measurement involved

5. Operating conditions

Description of the laser and pumping conditions
Continuous-wave, pulsed, temperature of operation

☐ Yes
☒ No

No laser measurement involved

Threshold values provided as density values (e.g. W cm^{-2} or J cm^{-2}) taking into account the area of the device

☐ Yes
☒ No

No laser measurement involved

6. Alternative explanations

Reasoning as to why alternative explanations have been ruled out as responsible for the emission characteristics
e.g. amplified spontaneous, directional scattering; modification of fluorescence spectrum by the cavity

☐ Yes
☒ No

No laser measurement involved

7. Theoretical analysis

Theoretical analysis that ensures that the experimental values measured are realistic and reasonable
e.g. laser threshold, linewidth, cavity gain-loss, efficiency

☐ Yes
☒ No

No laser measurement involved

8. Statistics

Number of devices fabricated and tested

☐ Yes
☒ No

No laser measurement involved

Statistical analysis of the device performance and lifetime (time to failure)

☐ Yes
☒ No

No laser measurement involved

March 2018
